# Detection of herpes simplex viruses in the oral lesions of patients with pemphigus vulgaris: Is it diagnostic or predictive of disease severity?

**DOI:** 10.1002/ski2.261

**Published:** 2023-06-19

**Authors:** Kambiz Kamyab, Maryam Daneshpazhooh, Nooshin Zaresharifi, Alireza Ghanadan, Reza Shahsiah, Hamid Reza Mahmoudi, Shirin Zaresharifi

**Affiliations:** ^1^ Department of Dermatopathology Tehran University of Medical Sciences Tehran Iran; ^2^ Department of Dermatology Tehran University of Medical Sciences Tehran Iran; ^3^ Department of Pathology Guilan University of Medical Sciences Rasht Iran; ^4^ Cancer Research Centre Tehran University of Medical Sciences Tehran Iran; ^5^ Skin Research Center Shahid Beheshti University of Medical Sciences Tehran Iran

## Abstract

**Background:**

Some studies emphasise the relationship between the herpes simplex virus (HSV) and pemphigus. Although the possible role of HSV in the pathogenesis of pemphigus and the severity of the disease is obscure, we aimed to evaluate the presence of herpes simplex viruses (HSV 1/2) in the oral lesions of patients with pemphigus vulgaris and also assess its association with disease severity and types of lesions.

**Methods:**

A retrospective study was conducted on collected data in the form of collecting paraffin blocks, slides, and relevant pathology reports and referring to patients' medical records. A questionnaire containing details on the degree of skin, scalp, and mucosal involvement (Pemphigus Disease Area Index (PDAI)) was fulfiled. The immunoassay result was also collected to check the anti‐desmoglein 3 and 1 antibodies (using ELISA technique).

**Results:**

In this study, 52 patients of pemphigus vulgaris with oral lesions (case) and 52 patients with oral lesions not related to the disease (control) were evaluated. HSV1 was detected in 13.5% of oral pemphigus lesions and 1.9% of the control group (*p* = 0.0598). There were no positive cases of HSV2 in either group. There was no significant association between the positivity of HSV1 and the site of lesions (*p* = 1.00) or disease severity (*p* = 0.28). However, we found a strong correlation between the PDAI disease severity score with the titre of the AntiDsg3 antibody (*r* = 0.487, *p* = 0.001) and AntiDsg1 antibody (*r* = 0.309, *p* = 0.026).

**Conclusion:**

This study demonstrated a significant prevalence of HSV1 in oral pemphigus lesions, and acyclovir therapy may play a significant role in managing these patients. However, HSV's role in the *pathogenesis* of pemphigus vulgaris cannot be clearly determined.

## Graphical abstract

Controversial results exist upon the possible role of HSV in the pathogenesis of pemphigus and its severity. This study demonstrated a significant prevalence of HSV1 in oral pemphigus lesions, and acyclovir therapy may play a significant role in managing these patients.

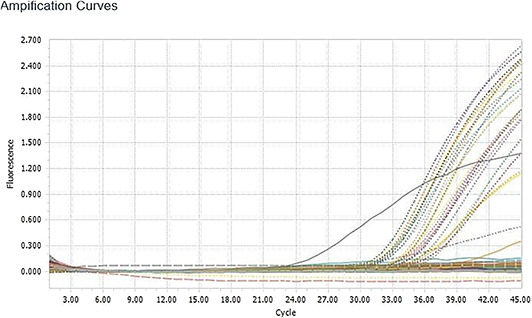

**What is already known about this topic?**

- Controversial results exist upon the possible role of HSV in the pathogenesis of pemphigus and its severity.

**What does this study add?**

- HSV may be present in oral lesions of immune‐suppressive therapy resistant Pemphigus.
- Acyclovir therapy may play a significant role in managing Pemphigus patients with oral lesions.

## 1 INTRODUCTION

Pemphigus vulgaris (PV) is an autoimmune bullous skin disease that is clinically characterised by blisters that form erosions on skin and mucosa.^1^ The characteristic histopathologic finding in PV is separation above the basal cell layer.^2^ The primary pathophysiological mechanism for PV lesions is the formation of autoantibodies, including antibodies against desmoglein 3 (Dsg 3) and desmoglein 1 (Dsg 1).^3^, ^4^ Dsg 3 and Dsg one involve in the cell‐to‐cell adhesion of keratinocytes; therefore, Dsg 1 and Dsg 3 antibodies can destroy intercellular desmosomal adhesive structures.^3^, ^4^ In this regard, changes in the expression of genes encoding these proteins are thought to be the primary cause of the development of these lesions. Furthermore, some environmental and even infectious factors have also been suggested to be effective in the occurrence of these lesions. Psychological stress, radiation, medications, malignancies, and even nutritional habits have been proven to play a role in the development and flare‐up of PV lesions.^5^, ^6^, ^7^, ^8^

Viral infections have been shown to stimulate disease‐related autoimmunity. In this context, the central role of herpes viruses in the incidence and exacerbation of PV has been widely studied.^9^, ^10^ For the first time, the close link between herpes simplex viruses (HSVs) and the risk for PV was described by Krain and colleagues in 1974. Subsequently, various studies have been conducted on the incidence and exacerbation of PV lesions following HSV infection. The possible relationship between HSV infection and PV lesions was suggested based on the elevated titres of HSV half IgG antibodies among PV patients.^11^ Based on the detection of HSV‐specific DNA in *swabs* taken from PV oral lesions, it was hypothesised that antiviral therapeutic regimens could improve the disease‐related lesions.^12^, ^13^ Furthermore, HSV infection was found to be related to exacerbation of PV manifestations.^10^, ^11^ Although previous studies have reported a promising role for HSV infection and PV, this hypothesis was not evaluated using gold‐standard methods, including *molecular* evaluation of PV *lesions*, in the majority of the studies.^11^, ^12^, ^13^ Furthermore, the findings of previous studies regarding the relationship between HSV and PV severity or exacerbation were contradictory.^5^, ^9^, ^10^, ^11^, ^12^, ^13^, ^14^ Since oral lesions are among the common findings in PV, identifying the presence of HSV infection in PV oral lesions can provide valuable justification for the pathogenesis of HSV infection in the formation of PV lesions. Therefore, the purpose of the present study was to evaluate the presence of HSV half in the oral lesions of patients with PV and to evaluate the diagnostic properties of HSV infection in oral lesions in the detection of PV.

## 2 MATERIALS AND METHODS

### 2.1 Study design

This case‐control study was conducted on paraffin blocks of patients with mucosal/mucocutaneous PV who were referred to the dermatology clinic of Razi Hospital, Tehran, Iran from 2016 to 2021. This study was approved by the Research Ethics Committee of the Tehran University of Medical Sciences and Razi Hospital (code: IR.TUMS.MEDICINE.REC.1399.214).

### 2.2 Study participants

The case group was selected among the PV patients who were referred to the mentioned hospital. The inclusion criterion for the case group was being documented new case of PV. The inclusion criteria for the control group were patients with oral lesions unrelated to PV, including oral erosion, oral lichen planus, margin of squamous cell carcinoma, and irritation fibroma. Patients in the control group were matched with the case group based on age and gender. Exclusion criteria for both the case and control groups were history of immunosuppressive therapy prior to diagnosis, or history of antiviral therapy.

Sample size was determined to be at least 50 participants in each group which considered a large sample size compared with similar previous studies. During the study period, 52 patients were included in each group.

### 2.3 Study procedure

#### 2.3.1 Instruments

A checklist was made to collect data from medical records, histopathological reports (suprabasal acantholysis, cleft), and direct immunofluorescence (IgG/C3 deposition in intercellular space, fishnet pattern) of the patients.

The Pemphigus Disease Area Index (PDAI) questionnaire was used to determine the degree of skin, scalp, and mucosal involvement. Disease activity is scored based on PDAI score in three domains scalp (10 points), skin (120 points) and mucosal activity (120 points) that sum up to the maximum 250 points. Higher PDAI scores indicate higher disease activity^15^ with the cut‐off values of 15 and 45, to distinguish moderate, significant, and extensive pemphigus forms.^16^ In this study, PDAI scores below and above 15 are defined as non‐severe and severe PV, respectively.

#### 2.3.2 Laboratory assessments

The enzyme‐linked immunosorbent assay (ELISA) technique was used to detect anti desmoglein 3 and 1 antibodies. The 20 RU/ml cut‐off was set to define positive results for these serum markers. Samples were examined using real‐time polymerase chain reaction (RT‐PCR) technique according to the manufacturer's instructions to determine HSV 1/2 viruses. DNA extraction was performed by columnar method using tissue extraction kit (Favorgene, Taiwan). The presence of HSV 1/2 virus genome was investigated using Geneproof kit (made in Germany) based on RT‐PCR method using the Lightcycler96 device. To evaluate the accuracy of tests in each run, the internal control (to check the presence of human cells and ensure the correct DNA extraction) positive control (to check the correct operation and sensitivity of the kits) non‐template control (NTC), and negative control (to check the possibility of contamination) were used.

#### 2.3.3 Statistical analysis

Normality of the data was evaluated using the Kolmogorov‐Smirnov test. The independent *t*‐test or Mann‐Whitney U tests were used to compare continuous variables between case and control groups for normally and non‐normally distributed variables, respectively. Chi‐Square or Fisher exact tests were used to evaluate the relationship between categorical variables. Pearson and Spearman correlation coefficients were used to evaluate the correlation between PDAI score and anti Dsg 1and anti Dsg 3 serum titres. *p* values less than 0.05 were considered statistically significant. Data analysis was performed using the statistical package for social sciences (SPSS) statistical software version 23.0 for windows (IBM, Armonk, New York).

## 3 RESULTS

In this study, 52 patients with oral PV lesions (case) and 52 patients with oral lesions unrelated to PV (control) were evaluated. Comparison of the demographic characteristics of the case and control groups is presented in Table 1. There was no significant difference between case and control groups in terms of age (*p* = 0.349) and gender (*p* = 0.432).

**TABLE 1:** Comparison of demographic characteristics between case and control groups.

Variable	Total	Case *n* = 52	Control *n* = 52	*p*
	*N* = 104			
Age (years)	45.8 ± 12.5	44.7 ± 10.4	47.0 ± 14.3	0.349^a^
Gender				
Male	49 (48.0%)	22 (42.3%)	27 (51.9%)	0.432^b^
Female	55 (52.0%)	30 (57.7%)	25 (48.1%)	

^a^ The independent *t*‐test was used to compare mean and standard deviation between groups.

^b^ The chi‐square test was used to compare the distribution pattern between case and control groups.

Overall, seven samples (13.5%) of the 52 paraffin blocks in the case group and 1 sample (1.9%) of paraffin blocks in the control group had a positive result for HSV1 (*p* = 0.0598). There were no positive cases of HSV2 in the two groups. The details of baseline characteristics and diagnostic parameters in those with HSV positivity in study and control groups are summarised in Table 2.

**TABLE 2:** The details of baseline characteristics and diagnostic parameters in those with HSV‐1 positivity in case and control groups.

	Group	Gender	Age	HSV2	Anti‐ Dsg1 antibody	Anti‐ Dsg3 antibody	Distribution	PDAI
1	Case	Female	45	Negative	3.00	>200	Mucosal	<15
2	Case	Male	44	Negative	4.00	>200	Mucosal	15–45
3	Case	Female	50	Negative	4.00	131	Mucosal	<15
4	Case	Male	51	Negative	23.50	138	Mucocutaneous	<15
5	Case	Male	51	Negative	6.00	>200	Mucosal	15–45
6	Case	Female	42	Negative	146	>200	Mucosal	15–45
7	Case	Female	52	Negative	13.00	>200	Mucocutaneous	15–45
8	Control	Female	33	Negative	–	–	–	–

**FIGURE 1:**
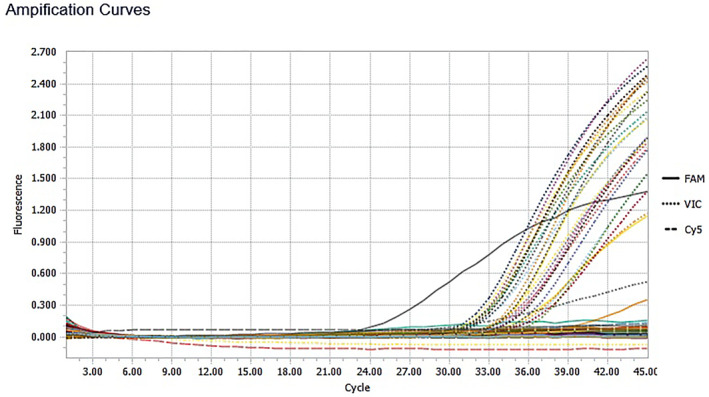
The diagram presents the results of a real time‐PCR run for the case group based on amplification curves. In this run, a positive example for HSV1 in cycle 24 (CT = 24) is observed (curves raised in cycles above CT 30 are related to positive and internal controls).

**FIGURE 2:**
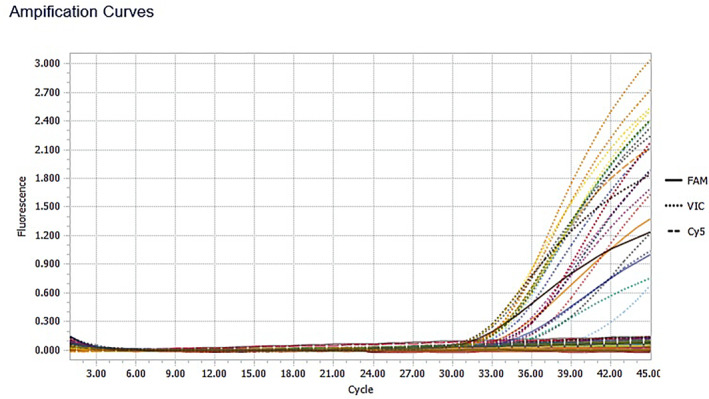
The chart shows the results of a real‐time‐PCR run for the control group based on amplification curves in which there was no positive HSV result.

The diagram of RT‐PCR run results based on amplification curves are demonstrated in Figure 1 (case group) and Figure 2 (control group).

Among HSV1 positive samples, 2 (28.6%) had mucocutaneous lesions and 5 (71.4%) had mucosal lesions. Among HSV1 negative samples 16 (35.6%) had mucocutaneous lesions and 29 (64.4%) had mucosal lesions. There was no significant association between the positivity of HSV1 and site of lesions (*p* = 0.999).

Relationship between PDAI scores and PV lesion location and HSV1 positivity is presented in Table 3. There was a significant relationship between disease severity (PDAI score) and the location of lesions (*p* = 0.040). Therefore, the likelihood of severe lesions was 4.08 times more in mucocutaneous than in mucosal sites. There was no significant relationship between PDAI score categories and HSV1 positivity (*p* = 0.285).

**TABLE 3:** Relationship between PDAI score categories and PV lesion dissemination and HSV1 positivity.

Variable	PDAI score		*p*
	<15	15–45	
PV lesion location			
Mucocutaneous	5 (27.8%)	13 (72.8%)	0.040^a^ ^,^ ^c^
Mucosal	21 (61.8%)	13 (38.2%)	
HSV1 positivity			
Positive	3 (42.8%)	4 (57.1%)	0.285^b^
Negative	27 (60.0%)	18 (40.0%)	

Abbreviations: HSV1, herpes simplex virus 1; PDAI, Pemphigus Disease Area Index; PV, pemphigus vulgaris.

^a^ The chi square test was used for the analysis.

^b^ The Fisher exact test was used for the analysis.

^c^ Significant relationship.

The median (IQR) for AntiDsg3 antibody titre in mucocutaneous and mucosal lesions were 200(0.0) and 200(73), respectively, which were not significantly different (*p* = 0.111). However, the median (IQR) level of AntiDsg1 antibody was significantly higher in mucocutaneous (50 [90.63]) compared to mucosal lesions (6.45 [13.25]) (*p* = 0.002).

Overall, we found a strong correlation between PDAI score and AntiDsg3 (*r* = 0.487, *p* = 0.001) and AntiDsg1 (*r* = 0.309, *p* = 0.026) antibody titres.

There was no difference in the median (IQR) of AntiDsg3 antibody titre between HSV1 positive and negative patients (200 [62] vs. 200 [43], respectively, *p* = 0.976). Similarly, there was no significant difference in the median (IQR) of AntiDsg1 antibody titres between HSV1 positive and negative patients (6 [19.5] vs. 13 [39.1], respectively, *p* = 0.830).

In general, the rate of HSV1 positivity was 13.6% in men and 13.3% in women indicating no difference between the two genders (*p* = 0.999). Also, the mean age of cases with and without HSV1 positivity was 47.85 ± 4.06 years and 44.15 ± 10.98 years, respectively, which were not significantly different (*p* = 0.112).

## 4 DISCUSSION

Environmental factors, including viral infections, along with genetic predisposition have an undeniable role in the *development* and *exacerbation* of autoimmune diseases. Among the viral infections, the role of HSV in the development of PV has been highlighted. The reason for such claims was based on the high titre of serum anti‐herpes antibodies as well as the detection of the virus genome in a number of disease‐related samples including tissue (mostly skin biopsies) and body fluid (saliva, …) specimens. However, this hypothesis has not yet been fully documented by evaluating HSV infection in immunocompetent PV patients using reliable gold standard methods in a large sample size and control group. Furthermore, the findings of previous studies on the relationship between HSV infection and PV were controversial.^10^, ^11^, ^12^, ^13^ In the present study, we evaluated HSV positivity in *oral mucosal lesions* of patients with new onset PV using the molecular gold standard method (PCR) in comparison with the control group consisting of a wide range of PV‐unrelated oral mucosal lesions. We also evaluated the relationship between HSV and disease severity in the present study.

Although the association between the oral lesions severity and HSV positivity was not statistically significant, considering the low power of the test (0.273), it can be estimated that this relationship may not stay non‐significant if the sample size is increased. Similar to the findings of the present study, Konda et al. reported HSV infection in 38.3% of PV patients and reported that HSV was associated with clinical and pathological features of PV.^11^ In the mentioned study, the presence of fissures, haemorrhagic crusts, erosions with angulated margins, linear erosions, and raised ESR were found to be significantly associated with HSV infection.^11^ In another study, PV patients carried significantly higher levels of anti‐HSV1 antibodies than healthy controls, and this effect was more pronounced in the active phase of the disease when compared to remission.^17^ Patients in the mentioned study were treated with immunosuppressive drugs, and the treatment may have a role in the activation of virus and high antibody titres due to previous infections.^17^ In another study, HSV infection and cessation of smoking were found to be correlated in oral pemphigus vulgaris lesions.^5^ In contrast to the findings of the present study, Mohammadi et al. did not find any association between HSV infection and PV.^14^

In this study, the location of the lesions (mucocutaneous vs. mucosal) and PDAI score, as two important indicators for disease severity, were not significantly related to HSV1 positivity. Therefore, the role of this virus in the disease severity cannot be confirmed.

Considering the contradictory findings of previous studies,^9^, ^10^, ^11^, ^12^ it is not yet clear whether tracking HSV genome in tissue or evaluating serum levels of HSV antibodies can predict PV severity. Nevertheless, what we found in our study was that the existence of HSV genome in oral lesions could not be a predictor of PV severity. Furthermore, it can be hypothesised that the presence of HSV in PV oral lesions might be the result of HSV activation due to tissue trauma caused by PV erosions. Regardless, as indicated in previous studies^18^ immunosuppressive therapy‐unresponsive pemphigus patients may benefit from oral acyclovir therapy.

### 4.1 Strengths and limitations

To the best of our knowledge this study was among the few studies that evaluated the presence of HSV *genome* in *oral mucosal lesions* of patients with PV, in particular, based on DNA extraction and polymerase chain reaction method. Although, using this gold standard technique, rather low prevalence of PV, and financial limitations prevented us from increasing the sample size, to the best of our knowledge, this study was the largest study on its kind that has been conducted on the oral lesions of PV patients and HSV. One of the limitations of this study might be the small number of positive HSV patients in our sample, which might have resulted in the non‐significant relationship between HSV positivity and PV severity. Although considering the low power of the test and the non‐significant result, it can be hypothesised that there is a high probability that the relationship will become significant by increasing the sample size. Therefore, conducting a study with larger sample size might result in a significant relationship between HSV one positivity and PV severity. Furthermore, considering the higher sensitivity of RT‐PCR in detecting HSV infection in fresh samples compared to formalin‐fixed paraffin‐embedded blocks, it can be suggested that further studies use *fresh samples* to evaluate the presence of HSV genome in oral PV lesions. Since all of the case samples were new cases with no prior history of treatment, immunosuppressive therapy cannot be suggested as a reason for virus activation. However, the history of viral infections along with other risk factors for PV should be included in discussing the findings.

## 5 CONCLUSION

Although HSV traces were present in a considerable number of oral PV lesions, no statistically significant relationship was observed between PV severity and HSV positivity. However, this finding can still indicate that HSV may be a risk factor for PV. More studies are needed to prove the causal relationship between HSV and oral lesions in PV. Although the role of HSV‐2/1 in the pathogenesis of PV is still debated, HSV1/2 may be present in oral lesions that are resistant to immune‐suppressive therapy.

## CONFLICT OF INTEREST STATEMENT

The authors declare no conflicts of interest.

## AUTHOR CONTRIBUTIONS

**Kambiz Kamyab**: Conceptualization (Equal); Data curation (Equal); Funding acquisition (Equal); Investigation (Equal); Methodology (Equal); Project administration (Equal); Resources (Equal); Supervision (Equal); Validation (Equal). **Maryam Daneshpazhooh**: Conceptualization (Equal); Data curation (Equal); Funding acquisition (Equal); Methodology (Equal); Project administration (Equal); Resources (Equal); Supervision (Equal); Validation (Equal). **Nooshin Zaresharifi**: Conceptualization (Equal); Data curation (Equal); Funding acquisition (Equal); Investigation (Equal); Methodology (Equal); Project administration (Equal); Resources (Equal); Writing – original draft (Equal); Writing – review & editing (Equal). **Alireza Ghanadan**: Data curation (Equal); Resources (Equal); Supervision (Equal); Validation (Equal). **Reza Shahsiah**: Conceptualization (Equal); Formal analysis (Equal); Methodology (Equal); Project administration (Equal); Software (Equal); Supervision (Equal); Validation (Equal). **Hamid Reza Mahmoudi**: Data curation (Equal); Resources (Equal); Supervision (Equal); Validation (Equal). **Shirin Zaresharifi**: Conceptualization (Equal); Supervision (Equal); Validation (Equal); Writing – review & editing (Equal).

## ETHICS STATEMENT

This study was approved by the Research Ethics Committee of the Tehran University of Medical Sciences and Razi Hospital (code: IR.TUMS.MEDICINE.REC.1399.214).

## Data Availability

The data that support the findings of this study are available from the corresponding author upon reasonable request.

## References

[1] Didona D , Paolino G , Di Zenzo G , Didona B , Pampena R , Di Nicola MR , et al. Pemphigus vulgaris: present and future therapeutic strategies. Dermatol Pract Concept. 2022;12(1):e2022037. 10.5826/dpc.1201a37 35223181 PMC8824520

[2] Kridin K , Schmidt E . Epidemiology of pemphigus. JID Innov. 2021;1(1):100004. 10.1016/j.xjidi.2021.100004 34909708 PMC8659392

[3] Malik AM , Tupchong S , Huang S , Are A , Hsu S , Motaparthi K . An updated review of pemphigus diseases. Medicina. 2021;57(10):1080. 10.3390/medicina57101080 34684117 PMC8540565

[4] Subadra K , Sathasivasubramanian S , Warrier A . Oral Pemphigus vulgaris. Cureus. 2021;13(9). 10.7759/cureus.18005 PMC8521543 34671517

[5] Bakhshi M , Manifar S , Azizi N , Asayesh H , Mansouri P , Nasiri S , et al. Risk factors in patients with oral pemphigus vulgaris: a case‐control study. Gen Dent. 2016;64(3):e10–e13. 27148665

[6] Costan V‐V , Popa C , Hâncu MF , Porumb‐Andrese E , Toader MP . Comprehensive review on the pathophysiology, clinical variants and management of pemphigus. Exp Ther Med. 2021;22(5):1–13. 10.3892/etm.2021.10770 PMC8495539 34630689

[7] Kridin K , Zelber‐Sagi S , Bergman R . Risk factors for lethal outcome in patients with pemphigus: a retrospective cohort study. Eur J Dermatol. 2018;28:26–37. 29521637 10.1684/ejd.2018.3252

[8] Vodo D , Sarig O , Geller S , Ben‐Asher E , Olender T , Bochner R , et al. Identification of a functional risk variant for pemphigus vulgaris in the ST18 gene. PLoS Genet. 2016;12(5):e1006008. 10.1371/journal.pgen.1006008 27148741 PMC4858139

[9] Baum S , Atar I , Coster D , Dovrat S , Solomon M , Sprecher E , et al. Relationship between pemphigus vulgaris severity and PCR‐positive herpes simplex virus. Acta Derm Venereol. 2022;102:adv00703. 10.2340/actadv.v102.917 35393625 PMC9631263

[10] Nili A , Karimi S , Salehi Farid A , Molhem Azar P , Farimani Z , Shahbazian H , et al. Factors associated with the healing time of pemphigus vulgaris oral lesions: a prospective study. Oral Diseases; 2022. 10.1111/odi.14236 35506253

[11] Konda D , Chandrashekar L , Dhodapkar R , Ganesh RN , Thappa DM . Clinical markers of herpes simplex virus infection in patients with pemphigus vulgaris. J Am Acad Dermatol. 2023;88(3):587–592. PMID: 31195023. 10.1016/j.jaad.2019.06.002 31195023

[12] Vega‐Memíje ME , García‐Vázquez FJ , Cuevas‐González JC , Rodríguez‐Lobato E , Aguilar‐Urbano MA . Is there a causal relationship between HSV‐1 and pemphigus vulgaris? SpringerPlus. 2015;4:1–3. 10.1186/s40064-015-1414-8 26722631 PMC4689730

[13] Zhang H , Wang Y , Li S , Yu M , Feng S . Prevalence and clinical features of herpes simplex virus infection in oral lesions of pemphigus vulgaris: a prospective, cross‐sectional study. J Am Acad Dermatol. 2022;87(5):1201–1203. 10.1016/j.jaad.2022.03.015 35283244

[14] Mohammadi F , Khalili Z , Marashi SM , Ehsani A , Daneshpazhooh M , Teymoori‐Rad M , et al. The potential roles of herpesvirus and cytomegalovirus in the exacerbation of pemphigus vulgaris. Dermatol Pract Concept. 2018;8(4):262–271. 10.5826/dpc.0804a03 30479853 PMC6246069

[15] Krain RL , Kushner CJ , Tarazi M , Gaffney RG , Yeguez AC , Zamalin DE , et al. Assessing the correlation between disease severity indices and quality of life measurement tools in pemphigus. Front Immunol. 2019;10:2571. 10.3389/fimmu.2019.02571 31781098 PMC6851056

[16] Boulard C , Duvert Lehembre S , Picard‐Dahan C , Kern JS , Zambruno G , Feliciani C , et al. Calculation of cut‐off values based on the Autoimmune Bullous Skin Disorder Intensity Score (ABSIS) and Pemphigus Disease Area Index (PDAI) pemphigus scoring systems for defining moderate, significant and extensive types of pemphigus. Br J Dermatol. 2016;175(1):142–149. 10.1111/bjd.14405 26800395

[17] Senger P , Abidi N , Lin DM , Seiffert‐Sinha K , Sinha AA . Positive correlation of anti‐herpes simplex type I virus antibody levels with pemphigus vulgaris disease status and activity in a large patient cohort. Eur J Dermatol. 2017;27(2):132–138. 10.1684/ejd.2016.2947 28174139

[18] Iraji F , Faghihi G , Siadat AH . The efficacy of acyclovir in treatment of the pemphigus vulgaris. J Res Med Sci. 2013;18(11):976–978. PMID: 24523784; PMCID: PMC3906789. 24523784 PMC3906789

